# Maternal and Neonatal Outcomes in Pregnant Women With SARS-CoV-2 Infection Complicated by Hepatic Dysfunction

**DOI:** 10.7759/cureus.25347

**Published:** 2022-05-26

**Authors:** Anisha Choudhary, Vinita Singh, Murari Bharadwaj

**Affiliations:** 1 Obstetrics and Gynecology, Tata Main Hospital, Jamshedpur, IND; 2 Family Medicine, Tata Main Hospital, Jamshedpur, IND

**Keywords:** hepatic dysfunction, maternal outcome, coronavirus, pregnancy, liver dysfunction, covid-19

## Abstract

Background and objective

The coronavirus disease 2019 (COVID-19) pandemic has caused one of the most devastating healthcare crises in recent times and presented many diagnostic challenges and uncertainties. COVID-19 complicated by acute hepatic dysfunction is a well-described phenomenon, but its impact on maternal and perinatal outcomes is not well documented. In this study, we aimed to evaluate the maternal and neonatal outcomes in pregnant women with COVID-19 complicated by liver dysfunction and compare those with pregnant women with COVID-19 and normal liver function.

Methodology

This was a retrospective observational cohort study conducted at the Tata Main Hospital, Jamshedpur, a tertiary care hospital in eastern India. All COVID-19-positive pregnant women (n=249) admitted to the hospital from May 15, 2020, to August 15, 2021, were included in this study. Retrospective data collection was done using the medical records of these COVID-19-positive pregnant women and included the baseline characteristics, past medical history, obstetric history, clinical presentation, laboratory results, management modalities, and maternal and neonatal outcomes. Of note, 107 women were found to have acute liver function abnormality on admission and 142 women had normal liver function tests (LFTs). Pregnant women with normal LFTs were classified as group one and those with deranged LFTs as group two. Characteristics such as age, period of gestation, symptoms, associated comorbidities, laboratory results, management, and outcomes were compared across both groups.

Results

Out of the total 249 pregnant women with COVID-19 admitted during the study period, 42.97% (n=107) women had laboratory findings consistent with liver dysfunction and 142 women (57.03%) had a normal liver function. Significantly higher levels of lactate dehydrogenase (LDH), C-reactive protein (CRP), neutrophil-lymphocyte ratio (NLR), alanine transaminase (ALT), aspartate aminotransferase (AST), and total bilirubin levels were seen in pregnant women with hepatic dysfunction when compared to those with normal liver function. Among the 249 patients, the majority were asymptomatic or had mild disease, 12 women had moderate disease, and six women had severe COVID-19. All women with severe COVID-19 had deranged LFTs. There was no statistical difference in terms of obstetric management between pregnant patients with and without liver dysfunction. Out of the 107 women with deranged liver function, 18 women had a preterm birth, four had intrauterine fetal death, and one had neonatal death. Complications such as postpartum hemorrhage, the need for blood transfusions, sepsis and multiorgan failure, and mortality were more commonly seen in the group of pregnant women with hepatic dysfunction associated with COVID-19.

Conclusion

COVID-19 in pregnancy may cause deranged LFTs in these women. Pregnant women with COVID-19 complicated by liver dysfunction have been reported to have worse inflammation, higher disease severity, and more morbidity and mortality when compared to those without liver dysfunction. They are also at a higher risk of complications such as postpartum hemorrhage, the need for blood transfusion, sepsis, and multiorgan dysfunction.

## Introduction

The ongoing coronavirus disease 2019 (COVID-19) pandemic presents an unprecedented challenge to public healthcare systems globally. It was first reported in December 2019 as a series of pneumonia-like cases in Wuhan, China [1]. Apart from the conventional involvement of the respiratory system resulting in symptoms like fever, cough, and dyspnea, an increasing number of patients have reported the involvement of other systems such as cardiovascular, gastrointestinal, hepatobiliary, renal, hematological, neurological, and ocular systems [2,3].

Several studies have reported liver dysfunction in patients with COVID-19; however, the degree and extent of the injury have not been well defined. Chen et al. have reported varying degrees of liver function abnormality in 43.4% of patients with severe acute respiratory syndrome coronavirus 2 (SARS-CoV-2) infection [4]. In another study from China, clinical records and laboratory results were obtained from 417 patients with COVID-19. They reported that 318 (76.3%) patients had abnormal liver function tests (LFTs) [5]. However, all these reports involved the non-pregnant adult population, and data on pregnant women with COVID-19 has been scarce.

Abnormal LFTs can be seen in 3-5% of pregnancies, with many potential causes, including preeclampsia; acute fatty liver of pregnancy (AFLP); viral hepatitis; cholestasis of pregnancy; or hemolysis, elevated liver enzymes, and low platelet count (HELLP) syndrome [6]. Deng et al. reported a 29.7% prevalence of liver injury in pregnant women with COVID-19 [7], whereas Chen et al. reported that 23.8-44.4% of pregnant women with SARS-CoV-2 infection had liver injury [8]. Determining the exact etiology of hepatic dysfunction in pregnant women with SARS-CoV-2 infection is particularly challenging due to the other competing diagnoses in the pregnant population.

Liver dysfunction during pregnancy can result in significant maternal and perinatal morbidity [6,9]. Hepatic dysfunction due to COVID-19 and preeclampsia may also have a synergistic effect leading to severe clinical manifestations as reported by Ahmed et al. [10]. To the best of our knowledge, there is no existing research from India on the effect of hepatic dysfunction due to SARS-CoV-2 infection on maternal and neonatal outcomes. In light of this, we aimed to analyze the prevalence of acute hepatic dysfunction as a complication in pregnant women with COVID-19 and the course and outcome of such pregnancies.

## Materials and methods

Study design and setting

This was a retrospective observational cohort study of pregnant women with SARS-CoV-2 infection admitted to the Tata Main Hospital, a tertiary care hospital in Jamshedpur, Jharkhand, India from May 15, 2020, to August 15, 2021. Liver function abnormality was defined as the elevation of either of the following liver enzymes in serum: alanine transaminase (ALT) >40 U/L, aspartate aminotransferase (AST) >40 U/L, and total bilirubin >17.1 μmol/L [5,11].

Data collection

All pregnant women visiting the hospital were tested for SARS-CoV-2 infection as per the national testing guidelines [12], and 249 pregnant women who tested positive for COVID-19 by reverse transcription-polymerase chain reaction (RT-PCR) or rapid antigen test (RAT) were included in the study. Patients who refused to be admitted or were advised to undergo home isolation were not included in this study.

Retrospective data collection was done using the medical records of these pregnant women and included the baseline characteristics, past medical history, obstetric history, clinical presentation, laboratory results, management modalities, and maternal and neonatal outcomes. Of note, 107 women were found to have acute liver function abnormality on admission and 142 women had normal LFTs. Pregnant women with normal LFTs were classified as group one and those with deranged LFTs as group two. Characteristics such as age, period of gestation, symptoms, associated comorbidities, laboratory results, management, and outcome were compared across both groups.

Statistical analysis

Statistical analysis was performed with SPSS Statistics version 22.0 (IBM, Armonk, NY). Continuous variables were expressed as mean, median, and standard deviation and compared across the groups using the Mann-Whitney U test. Categorical variables were expressed as numbers and percentages of patients and compared across the groups using Pearson’s chi-square test for independence of attributes or Fisher's exact test as appropriate. An alpha level of 5% was taken, and a p-value <0.05 was considered statistically significant.

## Results

A total of 249 pregnant women with SARS-CoV-2 infection were included in this study. Of these, 107 (42.97%) women had laboratory findings consistent with liver dysfunction and 142 women (57.03%) had normal ALT, AST, and total bilirubin levels. As shown in Table 1, among the patients with liver dysfunction, the mean age was 28.38 years, and it was 27.51 years among those without liver dysfunction. The mean period of gestation for the group with liver dysfunction was 35.42 weeks.

**Table 1:** Demographic profile of pregnant women with SARS-CoV-2 infection SARS-CoV-2: severe acute respiratory syndrome coronavirus 2

Parameter	Pregnant women without liver dysfunction (n=142)			Pregnant women with liver dysfunction (n=107)			P-value
	Mean	Median	Standard deviation	Mean	Median	Standard deviation	
Age (years)	27.51	27.00	4.42	28.38	28.00	4.58	0.058
Period of gestation (weeks)	34.49	37.20	7.81	35.42	37.20	6.24	0.672

Compared to pregnant women without liver injury, those with liver injury had significantly higher levels of lactate dehydrogenase (LDH), C-reactive protein (CRP), neutrophil-lymphocyte ratio (NLR), ALT, AST, and total bilirubin levels. Women with liver dysfunction had higher levels of ferritin and lower levels of platelet count when compared to those with normal LFTs. The prothrombin time was significantly higher in the group of pregnant women with liver dysfunction (Table 2).

**Table 2:** Laboratory investigations of pregnant women with SARS-CoV-2 infection *Significant p-value at <0.05 SARS-CoV-2: severe acute respiratory syndrome coronavirus 2; T.BIL: total bilirubin; ALT: alanine transaminase; AST: aspartate aminotransferase; LDH: lactate dehydrogenase; CRP: C-reactive protein; Hb: hemoglobin; TLC: total leukocyte count; NLR: neutrophil-lymphocyte ratio; PT: prothrombin time

Parameter	Pregnant women without liver dysfunction (n=142)			Pregnant women with liver dysfunction (n=107)			P-value
	Mean	Median	Standard deviation	Mean	Median	Standard deviation	
T.BIL (mg/dl)	0.56	0.53	0.19	0.93	0.68	0.91	<0.001^*^
ALT (U/I)	21.63	21.50	8.10	178.05	117.00	160.76	<0.001^*^
AST (U/I)	28.93	29.00	7.68	193.64	127.00	200.19	<0.001^*^
Serum ferritin (ng/ml)	43.48	38.00	22.72	46.32	38.90	34.42	0.720
LDH (U/I)	276.69	186.00	223.70	360.05	300.00	266.09	<0.001^*^
CRP (mg/L)	4.24	0.90	5.55	16.94	3.18	79.90	0.006^*^
Hb (gm/dl)	11.25	11.30	1.35	10.66	10.90	1.73	0.027
TLC (cells per mm^3^)	12,385.92	9,650.00	16,813.56	16,310.28	9,900.00	26,361.62	0.188
NLR	5.70	3.95	4.94	6.37	5.29	4.08	0.004^*^
Platelet (cells per mm^3^)	159,028.17	148,000.00	61,609.49	155,850.47	129,000.00	63,706.99	0.417
PT (seconds)	10.75	10.70	0.72	11.47	11.05	1.96	<0.001^*^
Serum creatinine (mg/dl)	0.64	0.64	0.14	0.70	0.64	0.32	0.211

As shown in Table 3, in the group of women with liver dysfunction, 17 women suffered from hypertensive disorders (preeclampsia, gestational hypertension, and chronic hypertension), 14 women had a history of diabetic disorders (gestational diabetes, diabetes mellitus type 2), 16 women had intrahepatic cholestasis of pregnancy (IHCP), and 14 women were diagnosed with anemia; however, these did not have any significant difference when compared to the group without liver dysfunction. None of the patients had a history of pre-existing liver disorders.

**Table 3:** Comorbidities present in pregnant women with SARS-CoV-2 infection SARS-CoV-2: severe acute respiratory syndrome coronavirus 2; DM: diabetes mellitus; GDM: gestational diabetes mellitus; GHTN: gestational hypertension; HTN: hypertension; IHCP: intrahepatic cholestasis of pregnancy

Parameter	Pregnant women without liver injury (n=142)		Pregnant women with liver injury (n=107)		P-value
	Number	Percentage	Number	Percentage	
DM/GDM	9	6.34	14	13.08	0.069
Cardiovascular diseases	1	0.70	1	0.93	0.840
Preeclampsia/GHTN/HTN	16	11.27	17	15.89	0.287
IHCP	15	10.56	16	14.95	0.285
Anemia	9	6.34	14	13.08	0.069

The severity of COVID-19 was categorized as per the clinical management protocol for COVID-19 in adults [13]. Of the total 249 women, 95 were asymptomatic, 136 had mild disease, 12 had moderate disease, and six women had severe COVID-19. There was a statistically significant difference in the disease severity between women with and without hepatic dysfunction (Table 4). A higher proportion of women with the asymptomatic or mild disease had normal liver enzymes whereas all women with severe disease had deranged LFTs. Of the six women with severe disease, four died due to the complications of severe COVID-19 pneumonia.

**Table 4:** Severity of COVID-19 among pregnant women *Significant p-value at <0.05 COVID-19: coronavirus disease 2019

Severity	Pregnant women without liver injury		Pregnant women with liver injury		P-value
	Number	Percentage	Number	Percentage	
Asymptomatic	68	47.89	27	25.23	<0.001^*^
Mild	69	48.59	67	62.62	
Moderate	5	3.52	7	6.54	
Severe	0	0	6	5.61	
Total	142	100	107	100	-

There was no statistically significant difference in terms of obstetric management between pregnant patients with and without liver dysfunction (Table 5). A higher percentage of women had a cesarean section in the group with liver dysfunction when compared to the group with normal liver function; however, this difference was not statistically significant.

**Table 5:** Management of pregnant women with COVID-19 COVID-19: coronavirus disease 2019

Management	Pregnant women without liver injury		Pregnant women with liver injury		P-value
	Number	Percentage	Number	Percentage	
Conservative	13	9.15	15	14.02	0.155
Suction evacuation	2	1.41	2	1.87	
Hysterotomy	1	0.7	0	0	
Laparotomy	3	2.11	0	0	
Cesarean section	79	55.63	70	65.42	
Vaginal delivery	43	30.28	20	18.69	
Vaginal tear repair	1	0.7	0	0	
Total	142	100	107	100	-

Of the 107 women with deranged liver function, 18 had a preterm birth, four had intrauterine fetal death, and one had neonatal death. Three babies tested positive for SARS-CoV-2 infection within 24 hours of delivery, two of which belonged to women in the group with hepatic dysfunction (Table 6).

**Table 6:** Neonatal outcomes in pregnant women with COVID-19 COVID-19: coronavirus disease 2019; LBW: low birth weight; IUD: intrauterine fetal death; NND: neonatal death

Parameter	Pregnant women without liver injury		Pregnant women with liver injury		P-value
	Number	Percentage	Number	Percentage	
Preterm	38	31.15	18	20.22	0.076
LBW	39	32.50	23	26.44	0.347
IUD	2	1.41	4	3.81	0.406
NND	3	2.50	1	1.12	0.638
Baby COVID-19-positive	1	50.83	2	2.25	0.576

There was a statistically significant difference in the complication rates between the two groups (Table 7). Postpartum hemorrhage, the need for blood transfusion, complications like sepsis and multiorgan failure, and mortality were more commonly seen in the group of pregnant women with hepatic dysfunction associated with SARS-CoV-2 infection.

**Table 7:** Maternal complications in women with COVID-19 *Significant p-value at <0.05 COVID-19: coronavirus disease 2019; MODS: multiorgan dysfunction syndrome; AFLP: acute fatty liver of pregnancy

Parameter	Pregnant women without liver injury		Pregnant women with liver injury		P-value
	Number	Percentage	Number	Percentage	
Postpartum hemorrhage	7	5.73	16	17.77	0.003^*^
Sepsis with MODS	0	0	4	3.74	0.033^*^
Blood transfusion	2	1.41	9	8.41	0.008^*^
AFLP	0	0	1	0.93	0.316
Maternal mortality	0	0	4	3.74	0.033^*^

## Discussion

Acute liver complications due to novel coronavirus infection are a well-described phenomenon. Liver dysfunction was also reported in the Middle East respiratory syndrome coronavirus (MERS-CoV) and SARS-CoV [14,15]. While the exact cause of hepatic injury is uncertain, several factors have been associated with the cause of elevated serum transaminase levels in SARS-CoV-2-infected patients. Both the liver and bile duct cells express angiotensin-converting enzyme 2 (ACE2) receptors that are the binding site for cellular entry by SARS-CoV-2, resulting in deranged LFTs [16,17]. Another hypothesis points to immune-mediated liver injury that is cytokine storm, causing the release of multiple proinflammatory cytokines, particularly interleukin-6 (IL-6), which causes both pulmonary and extrapulmonary damage. Other proposed mechanisms include severe hypoxemia due to acute respiratory failure, drug interactions, septic shock, and multiorgan dysfunction [18].

Philips et al. have reported that among patients who tested positive for SARS-CoV-2, 45% had mild, 21% had moderate, and 6.4% had severe liver injury [19]. They also reported that severe acute liver injury was significantly associated with elevated inflammatory markers and the patients with severe liver injury had a more severe clinical course, including higher rates of intensive care unit (ICU) admission (69%), intubation (65%), renal replacement therapy (33%), and mortality (42%). However, data regarding liver dysfunction in pregnant women with COVID-19 infection remains scarce.

Approximately 3-5% of women are reported to be diagnosed with liver dysfunction during pregnancy [6]. Certain liver diseases are uniquely associated with pregnancy, such as hyperemesis gravidarum, IHCP, AFLP, and HELLP syndrome. Another type is liver disease not related to pregnancy, such as viral or drug-induced hepatitis. Also, pregnant women may have pre-existing liver disorders such as cirrhosis and portal hypertension. Some of these conditions can be fatal to both the mother and the fetus.

Pregnant women with COVID-19 belong to a vulnerable group of the population due to concerns about the effect of the disease on the mother and the baby. As pregnancy is already an immunocompromised state, pregnant women with COVID-19 are at increased risk of admission to ICUs and requiring invasive ventilation [19]. Limited studies are available on the effect of hepatic dysfunction associated with SARS-CoV-2 infection on pregnancy outcomes. In our study, we found that 107 (42.97%) pregnant women with COVID-19 infection had deranged LFTs. Deng et al. conducted a similar study on a group of 37 pregnant women with COVID-19 and reported that 11 (29.7%) women had findings consistent with acute liver dysfunction [7], whereas Chen et al. reported that 23.8-44.4% of pregnant women with COVID-19 had liver injury [8]; however, they did not study the effect of liver dysfunction on pregnancy outcomes.

In our study, we compared the level of other inflammatory markers between the groups of pregnant women with and without liver injury. It was seen that compared to pregnant women without liver injury, those with liver injury had significantly higher levels of LDH, CRP, prothrombin time, and neutrophil-lymphocyte ratio. Deng et al. studied a total of 37 pregnant patients, and they found that inflammatory markers like CRP, LDH, procalcitonin, and IL-6 were higher in pregnant COVID-19-positive women with liver injury [7]. Similar associations between abnormal LFTs and elevated levels of inflammatory markers are reported in studies on non-pregnant adults with COVID-19 infection [19,20]. We also found that platelet level was lower in women with hepatic dysfunction, and more studies are required to gain deeper insights into the risk of thrombocytopenia and hemorrhage in pregnant women with liver dysfunction associated with COVID-19 infection.

There is growing evidence to suggest that increased systemic inflammation associated with liver dysfunction results in a higher incidence of severe COVID-19 and related mortality [19,21,22]. We also found a positive correlation between liver dysfunction and the severity of the disease. We had earlier compared the pregnancy outcomes in women with COVID-19 during the first and second waves of the pandemic at our institute and had found that the severity of the disease and frequency of hepatic dysfunction were significantly higher in the second wave when compared to the first [23]. Acute liver dysfunction was seen in all patients with severe COVID-19, suggesting that it is appropriate to monitor liver function in pregnant patients with COVID-19.

In our study, out of 107 pregnant women with liver dysfunction, 17 women suffered from hypertensive disorders (preeclampsia, gestational hypertension, and chronic hypertension), 14 women gave a history of diabetic disorders (gestational diabetes, diabetes mellitus type 2), 16 women had IHCP, and 14 women were diagnosed with anemia. There was no significant difference in the prevalence of associated comorbidities between the groups. Both preeclampsia and SARS-CoV-2 infection are examples of microvascular diseases that can cause endothelial injury. They both can have a synergistic effect and can cause a high prothrombotic tendency leading to multiorgan failure [10]. At our institute too, we had a pregnant woman with COVID-19 and preeclampsia complicated by AFLP and acute kidney injury [24]. Mendoza et al. reported that pregnant women with severe COVID-19 can develop a preeclampsia-like syndrome [25]. Preeclampsia and liver dysfunction during pregnancy can worsen due to SARS-CoV-2 infection and healthcare providers should be aware of it and monitor pregnancies with pre-existing comorbidities like preeclampsia with extra caution.

A higher percentage of women in the group with liver dysfunction had cesarean section when compared to the group with normal liver function; however, there was no statistically significant difference in overall obstetric management between pregnant patients with and without liver injury. Of the 107 women with deranged liver function, 18 women had a preterm birth, four had intrauterine fetal death, and one had neonatal death. Pregnant women with COVID-19 are more likely to deliver preterm and get admitted to the neonatal care units [26]. Similarly, in our study, of the total 249 pregnant women with SARS-CoV-2 infection, 56 (22.49%) women had a preterm birth, 18 of whom belonged to the group with hepatic dysfunction. Three babies tested positive for COVID-19 within 24 hours of delivery, two of which belonged to women in the group with hepatic dysfunction. Similar findings were reported by Deng et al. [7]. Contrary to our findings, Can et al. reported significantly low birth weight in pregnant women with COVID-19 complicated by hepatic dysfunction than those without liver dysfunction [27]. Larger studies are required to further explore the effect of liver dysfunction on neonatal outcomes.

In our study, we found a significantly higher rate of complication in the group with hepatic dysfunction when compared to the group with normal baseline LFTs. Postpartum hemorrhage, the need for blood transfusions, complications like sepsis and multiorgan failure, and mortality were more often seen in the group with hepatic dysfunction. A higher frequency of adverse maternal and fetal outcomes was observed in pregnant women with liver dysfunction [28]. Furthermore, deranged liver function was associated with severe COVID-19 with the extent of derangement being proportional to the severity of COVID-19 [29]. In their study, Can et al. also reported that pregnant women with COVID-19-associated liver dysfunction had an increased frequency of severe disease and longer hospital stay than those without liver dysfunction [27]. Hence, it can be concluded that pregnancy with COVID-19 complicated by liver dysfunction is associated with a higher risk of disease severity and other maternal complications. Liver function evaluation should be performed for all pregnant women with COVID-19, and clinicians should be extra vigilant in cases with abnormal LFTs and those with pre-existing comorbidities like preeclampsia.

This study has a few limitations. Some patients may have been unaware of the underlying liver disease and this may have led to an underestimation of pre-existing liver disease. We had done only baseline liver function tests on admission for the majority of these women; serial follow-up was not done to assess the progression of the dysfunction. As we are a referral center, many women were lost to follow-up, and hence long-term implications could not be studied. Lastly, our sample size was limited and from a single center. We believe that large-scale studies are required to further understand the full extent of the adverse effects of liver dysfunction due to COVID-19 on pregnancy outcomes.

## Conclusions

The results of the present study demonstrate that pregnant women with SARS-CoV-2 infection have a high incidence of liver dysfunction. Pregnant women with liver dysfunction have a higher incidence of severe disease, morbidity, and mortality. They suffer from worse inflammation than those without liver injury and are at a greater risk of complications like postpartum hemorrhage, need for blood transfusion, and multiorgan dysfunction. There was no evidence to indicate that liver dysfunction worsens neonatal outcomes. Women with pre-existing comorbidities like preeclampsia and liver disease have a higher chance of experiencing worsening disease conditions during pregnancy. Liver function should be monitored in all pregnant women with COVID-19 and clinicians should be aware of its implications on the disease course and pregnancy outcomes.
